# A Re-examination of the Measurement of Foot Strike Mechanics During Running: The Immediate Effect of Footwear Midsole Thickness

**DOI:** 10.3389/fspor.2022.824183

**Published:** 2022-04-26

**Authors:** Zhenyuan Zhang, Mark Lake

**Affiliations:** School of Sport and Exercise Science, Liverpool John Moores University, Liverpool, United Kingdom

**Keywords:** landing posture, landing dynamics, vertical loading rates, joint stiffness, shoe cushioning, impact phase

## Abstract

**Purpose:**

Midsole cushioning thickness (MT) is a key component of running footwear that may influence the stiffness setting of the joints, performance enhancement, and injury prevention. Most studies that have investigated the influence of manipulating shoe midsole characteristics on foot strike patterns and vertical force loading rates have not considered the dynamic conditions of initial landing and the associated initial lower limb joint stiffness. In this study, we examined the effect of running in shoes with large changes in MT on both the posture and dynamics associated with foot strike.

**Methods:**

12 injury-free runners with habitual rearfoot strike patterns ran at 4.5 m/s along a 40-m runway in shoe conditions with MT of 30, 42, and 54 mm, respectively. Ground reaction force and the right leg kinematic data were collected. One-way repeated measures ANOVA was conducted to statistically analyze the effect of MT on key variables linked to foot strike.

**Results:**

Increased midsole thickness resulted in a slightly flatter foot strike posture (*p* < 0.05), a decreased shank retraction velocity (*p* < 0.05), and an increase in forward horizontal foot velocity (*p* < 0.05), all at initial ground contact. Vertical force loading rates were reduced with increasing MT (*p* < 0.05), but this was associated with large increases in the initial ankle and knee joint stiffness (*p* < 0.05).

**Conclusion:**

Adjustments in the initial conditions of contact with the ground during running were seen in both the posture and dynamics of the lower limbs. To help to mitigate the impact severity from foot-ground collision with the thinnest shoe condition, there was an increased shank retraction velocity and decreased forward velocity of the foot at landing. These active impact-moderating adaptations likely served to reduce the changes in impact severity expected due to midsole material properties alone and should be considered in relation to altering the risk of running-related injuries.

## Introduction

Footwear cushioning can have a significant effect on the mechanics of running gait and foot strike patterns (Fuller et al., 2015). Cushioning properties can be influenced by midsole thickness (MT), and this aspect has received more attention recently with the sport governing body (World Athletics) amending footwear rules to limit the MT of running shoes to a maximum of 40 mm (World Athletics Organization, 2020). Burns and Tam (2020) proposed that the MT should be limited, as they assumed that the increased MT would result in advantages in running performance and effectively evolve running shoes into an unrecognizable extension of the human body. This assertion has provoked a debate on the regulation of competition running shoes, followed by the commentaries from Frederick (2020), Hoogkamer (2020), and Nigg et al. (2020) which argued that there was not sufficient evidence to support a performance advantage attributable to increased MT. However, alongside traditional running shoes, footwear manufacturers have begun to release ultra-cushioned shoes with exceptionally thick midsoles to the market. These shoes are designed to provide extra-cushioning by attenuating more of the impact from foot-ground collision during running (Sun et al., 2020). Lower vertical loading rates could be expected in a thicker midsole condition since the extra-cushioned heel allows more vertical deformation of the midsole materials and hence dampens impact loading from the foot-ground collision (Lieberman, 2012; Gruber et al., 2021), although a previous study indicated that the MT beyond 25 mm might not further reduce the vertical loading rates (Law et al., 2019). High loading rates have been associated with the risk of several running-related injuries including patellofemoral pain (Yang et al., 2019), tibial stress fracture (Pohl et al., 2008), and plantar fasciitis (Pohl et al., 2009) in habitual rearfoot strikers, although the evidence is inconclusive.

From an injury prevention perspective, different MT running footwears can indeed affect a few biomechanical parameters that may be linked with injury risk, such as lower limb posture at foot strike and vertical ground reaction force (GRF) loading rates (Sinclair et al., 2016). Previous studies reported that running shoes with increased MT promoted a more heel-first strike pattern at ground contact (Squadrone et al., 2015), but this study compared minimalist vs. conventional running footwear. A study of moderate MT changes in conventional footwear for rearfoot strike runners did not find large changes in the degree or severity of heel strike (Law et al., 2019). Adjustments in foot strike pattern due to MT modifications are likely dependent on the spectrum of MT examined. It has been assumed that heel strike landing is associated with a high vertical force loading rate. But recent, large sample size, cross-sectional studies found a non-linear relationship between foot strike angle (FSA) and peak vertical loading rate, with more severe heel strikes associated with lower vertical loading rates (Stiffler-Joachim et al., 2019; Van den Berghe et al., 2021). This was typified by the longitudinal case study of an ultra-distance runner which revealed that their extreme heel strike running style was associated with a low vertical force loading rate (Van den Berghe et al., 2021). The authors described the “soft” landing strategy of that runner and found that the touchdown foot velocity was 0.35 ms^−1^ lower than the average velocity for their large database of runners and this likely contributed to the reduced vertical force loading rate. Touchdown foot velocity during running was previously shown to be a very important determinant of both impact peak force and loading rate (Gerritsen et al., 1995). Using a direct dynamic simulation of heel strike running, it was found that every 0.1-ms^−1^ increase in touchdown velocity increased impact peak force by 212 N. These findings need to be confirmed with subject testing but despite many investigations of lower limb impact loading during running while manipulating footwear, very few investigations have measured or considered the dynamic conditions of landing. It has been found that in preparation for landing on a less cushioned surface, habitual rearfoot strikers decreased their heel velocity at the instant of landing during running (Dixon et al., 2005). The same strategy is possible during running in less cushioned shoes, but it remains to be fully explored.

The initial conditions of landing as the foot collides with the ground during running and the associated impact severity can be influenced by active adaptation in lower limb motion during the late swing phase. This can affect the dynamics of the ankle and knee joints and velocity or momentum of the foot at the initial contact (IC). The swing leg is moved rearward toward the ground (i.e., retraction) just prior to IC, and this has been shown to reduce horizontal foot velocity at landing and therefore impact severity during barefoot running (De Wit et al., 2000). Those authors also found that faster knee flexion occurred both prior to and at IC in barefoot running compared to shod running, which indicated an active mechanism to modify the dynamics of landing and reduce impact severity. Adjustments in landing dynamics (as well as posture) of the lower limbs at IC may be associated with the initial stiffness setting of the joints during early ground contact. Seyfarth et al. (2003) also documented that leg retraction was employed before ground contact. They found that during treadmill running, the magnitude and velocity of retraction prior to IC increased when the swing phase of running was perturbed, although the shank angle to the vertical at IC (referred to as the angle of attack) was unchanged. Therefore, it is likely that some adaptations in the mechanics of foot strike to footwear modifications such as increased MT could be reflected in the dynamics of the foot and lower leg rather than the posture at IC. As suggested earlier, despite the recent research interest in the mechanics of foot strike during running, few investigations have considered the initial dynamic conditions of landing. If the lower limb joints were actively flexing at the instant of ground contact, then that would also have implications for the joint stiffness setting at foot strike.

The stiffness of the lower limbs during running has been related to both performance (Butler et al., 2003) and running-related injuries (Messier et al., 2018). Therefore, it is worthwhile to examine how lower limb joint stiffness differs when running in shoes with different MTs. Based on the conventional measurement of joint stiffness (Hamill et al., 2014), the lower limb has been typically modeled as a torsional spring and the torsional stiffness for joints (also termed as “quasi-stiffness”) have been determined as a ratio of changes in joint moment to changes in joint angle during the energy absorption phase of landing. This energy absorption phase was operationally defined as the period from IC to the maximal joint angular flexion in mid-stance (Hamill et al., 2014; Borgia and Becker, 2019; Borgia et al., 2021). Using this stiffness calculation approach, Borgia and Becker (2019) found that shoes with ultra-thick cushioning resulted in a stiffer ankle but a more compliant knee joint, while a recent paper published from Gruber et al. (2021) reported no differences in lower limb joint stiffness between running in shoes with different cushioning or MT characteristics. The conflicting results were attributed to the differences in the running speed examined or modifications in the calculation of leg stiffness (Gruber et al., 2021). It is possible that the assumption of near constant joint stiffness (i.e., linear rotational spring) over the first half of stance may be over simplistic and therefore lacks sensitivity to changes that might occur due to footwear cushioning? Nigro et al. (2021) confirmed the non-linear nature of joint stiffness during running, but it is plausible that using a smaller duration of the stance phase, that includes just the initial impact phase (up to the timing of peak knee flexion velocity, which is usually after the first 50–60 ms of stance), the joint stiffness characteristics might be more linear and also better suited to distinguish between footwear conditions and, perhaps in turn, risk factors for injury. The initial loading and joint stiffness setting surrounding foot impact with the ground is of key importance regarding running-related injury risk (Milner et al., 2007). Early ground contact joint stiffness can be determined during the initial impact phase only using the timing of the impact force peak (Milner et al., 2007) or the time to peak knee flexion velocity (Verheul et al., 2017). This stiffness corresponding to the impact phase only potentially provides more detailed information regarding joint stiffness setting surrounding foot strike and would likely be a sensitive assessment of adaptations in running mechanics due to running in shoes with differing MTs and cushioning characteristics. Impact phase stiffness has been previously used as a sensitive indicator of adaptations in neuromuscular function during running that may result from either training status or muscle damage after downhill running (Dutto and Braun, 2004; Verheul et al., 2017), but remain to be explored from the context of running footwear modifications.

Therefore, this study aims to re-examine the measurement of foot strike mechanics during running by having a more complete assessment of the initial conditions of ground contact that includes both the landing posture of the lower limb (as typically done) and the dynamics (velocity) of the lower limb at the instant of contact, as well as the impact phase-specific lower limb joint stiffness. This approach was used to examine the influence of systematically modifying the MT of running shoes on the mechanics of foot strike. It was hypothesized that: (1) runners would adapt to the footwear with increased MT by changing both the posture of the foot at ground contact and its dynamics; (2) the increased MT would result in lower GRF loading rates for runners, as the combination of extra-cushioning and impact-moderating movement adaptation leads to a decrease in impact severity; (3) ultra-cushioned footwear would increase impact phase stiffness of the ankle but decrease stiffness of the knee joint.

## Methods

### Participants

A number of 12 recreational runners (age = 26.9 ± 11.0 years; height = 1.81 ± 0.05 m; body mass = 73.6 ± 8.3 kg; weekly running mileage = 28.7 ± 18.3 km) with a shoe size of UK 9.5 participated in this study. All participants were free of any lower limb injuries in the past 6 months before the data collection and were running with habitual rearfoot strike patterns. The study protocols were approved by the Liverpool John Moores University Ethics Committee. Written consent forms were received from each participant prior to the data collection.

### Experimental Setup

An 8-camera motion capture system (Oqus 300, Qualisys AB, Gothenburg, Sweden) was deployed around the middle of a 40 m runway to record three-dimensional kinematics of the right lower limb during running. A 0.9 m × 0.6 m force platform (9281B, Kistler AG, Winterthur, Switzerland) was embedded in the runway at the center of the capture volume. Sampling rates for kinematic and GRF data were set at 500 and 1,500 Hz, respectively. A total of two sets of TCI photogates (Brower Timing System, Draper, UT, USA) were set 5 m apart around the force platform to monitor the average running speed for each participant.

### Protocols

**Figure 1:**
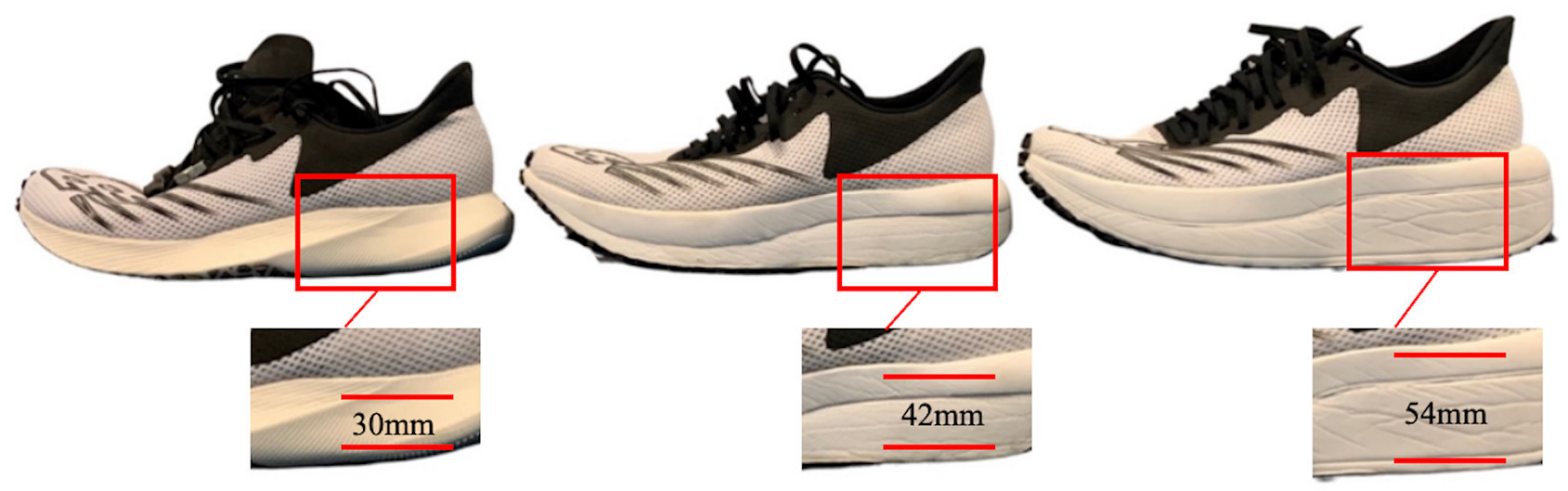
Three shoe models were tested in this study. From left to right, MT was 30, 42, and 54 mm, respectively.

There were 3 pairs of running shoes used in this study (Figure 1). Based on the mechanical reports from the shoe manufacturer, each shoe property was identical [e.g., shoe size (UK 9.5), shoe upper, outsole and midsole material] except the MT and, inevitably, the shoe mass. The MT was 30, 42, and 54 mm, respectively (Figure 1), and the corresponding shoe masses (averaged across each pair of shoes) were 221, 233, and 268 g, respectively, for the 30-, 42-, and 54-mm shoes. Initially, the masses of the shoes with substantially different MT were very different, and we did not want this to be a predominant factor in any comparison between shoes in terms of foot strike biomechanics. Therefore, we tried to keep the mass similar across shoe conditions by adding mass to the two lighter shoes. This was accomplished by threading lead weights to their lacing systems (Hoogkamer et al., 2016) and placing a thin, flexible layer of lead encapsulated in rubber beneath each insole. MT30, MT42, and MT54 were used to describe the corresponding shoe conditions. There was also a small difference in the heel to toe drop between shoes of 7 (MT30), 5 (MT42), and 3 (MT54) mm, respectively. This selection was designed to examine running foot strike mechanics using footwear with MT above and below the current limit imposed by World Athletics (40 mm). Retro-reflective markers (12 mm diameter) were attached on the locations of the 2nd metatarsal head [securely mounted on the shoe with glue (Super Glue, Loctite Inc.) after palpation of the anatomical location], medial and lateral malleoli, medial and lateral femoral epicondyle, and greater trochanter to define the foot, shank, and thigh segments of the right leg. Additional tracking markers were glued on each right shoe at the locations of the 1st and 5th metatarsal bases and the medial, lateral, and rear parts of the heel counter, respectively. Curved, lightweight carbon fiber plates with four non-collinear tracking markers were attached with bandages and tape to the lateral aspect of the right thigh and shank. A static standing calibration trial was first recorded in each shoe condition to determine the relative location of tracking markers and segment defining markers that were used for the kinematic model of the right lower limb for each participant. Participants warmed up by running along the runway for at least 5 min in each shoe condition before data collection. They were instructed to run along the runway in all three shoe conditions presented in a mixed order. For the three shoes, there were six different sequences for shoe presentation order, and these were repeated two times for the 12 subjects. A total of five successful trials for each shoe condition were collected based on the following criteria: (1) the running speed was within 4.5 m/s ± 5%; (2) their right foot contacted near the center of the force platform in the middle of the runway; (3) there was no adjustment in their natural stride pattern before and after contacting the force platform. To avoid any effects of fatigue, participants were allowed a rest between shoe conditions.

### Data Reduction

The three-dimensional marker trajectories were tracked using Qualisys Track Manager software (QTM 2020.1, Qualisys AB, Gothenburg, Sweden) and exported to Visual 3D software (C-Motion, MD, USA) for the following processing and analysis. GRF data were filtered by a 4th order, zero lag, low-pass Butterworth filter with a cutoff frequency of 50 Hz. To avoid over-smoothing high-frequency kinematic data of the foot and the noise associated with damped vibration of soft tissues of the calf and thigh on landing, the same filter with two different cutoff frequencies was applied to tracking markers on the shoe, shank, and thigh segments (Davis and Challis, 2021). Specifically, markers on the shoe were filtered at 25 Hz, and shank and thigh markers were filtered at 15 Hz (Mai et al., 2019). Pilot work had revealed that foot segment movements immediately after landing during running had high-frequency content, and, in an attempt to preserve this content, it was filtered separately using a higher filter cutoff frequency of 25 Hz (refer to Davis and Challis, 2021). The rigid attachment of the markers on the shoes reduced their oscillation due to running impacts.

The event of IC and toe-off for each running trial was determined using a vertical GRF threshold of 20 N. Joint angle and angular velocity at the right ankle and knee were determined by an X-Y-Z Cardan rotation sequence, which represented flexion or extension, abduction or adduction, and axial rotation. The current investigation focused on sagittal plane kinematic data only. For variables prior to IC (i.e., late swing phase), the timing and range of any backward angular displacement of the shank (retraction) were recorded. In this time period, knee joint motion was predominantly governed by the movement of shank segments. At the instant of IC, traditional foot strike “patterns” were determined using the FSA established in previous work (Zhang et al., 2017). FSA was computed by deducting the ankle angle during standing from the ankle angle at the IC to the ground. Subsequently, based on the previous literature (Altman and Davis, 2012), foot strike patterns were classified according to the following criteria: forefoot strike < -1.6° < midfoot strike <8° < rearfoot strike. Supplementary to FSA, shank angle to the vertical was also calculated as the angle between the shank segment and the laboratory vertical axis (Squadrone et al., 2015), since this factor may be associated with running performance (Folland et al., 2017). In addition, initial conditions of landing from a dynamic perspective were described by the retraction angular velocity and heel velocity for each shod condition (similar to De Wit et al., 2000). The horizontal and vertical components of heel velocity were calculated using the first derivate of target signals on Y- and Z-axes of the tracking marker placed on the rear of the heel counter. To reduce measurement error when estimating dynamic variables at a specific event in time (i.e., IC), horizontal and vertical components of heel velocity during the last five data points until IC (10 ms) were averaged to represent heel velocity at IC.

**Figure 2:**
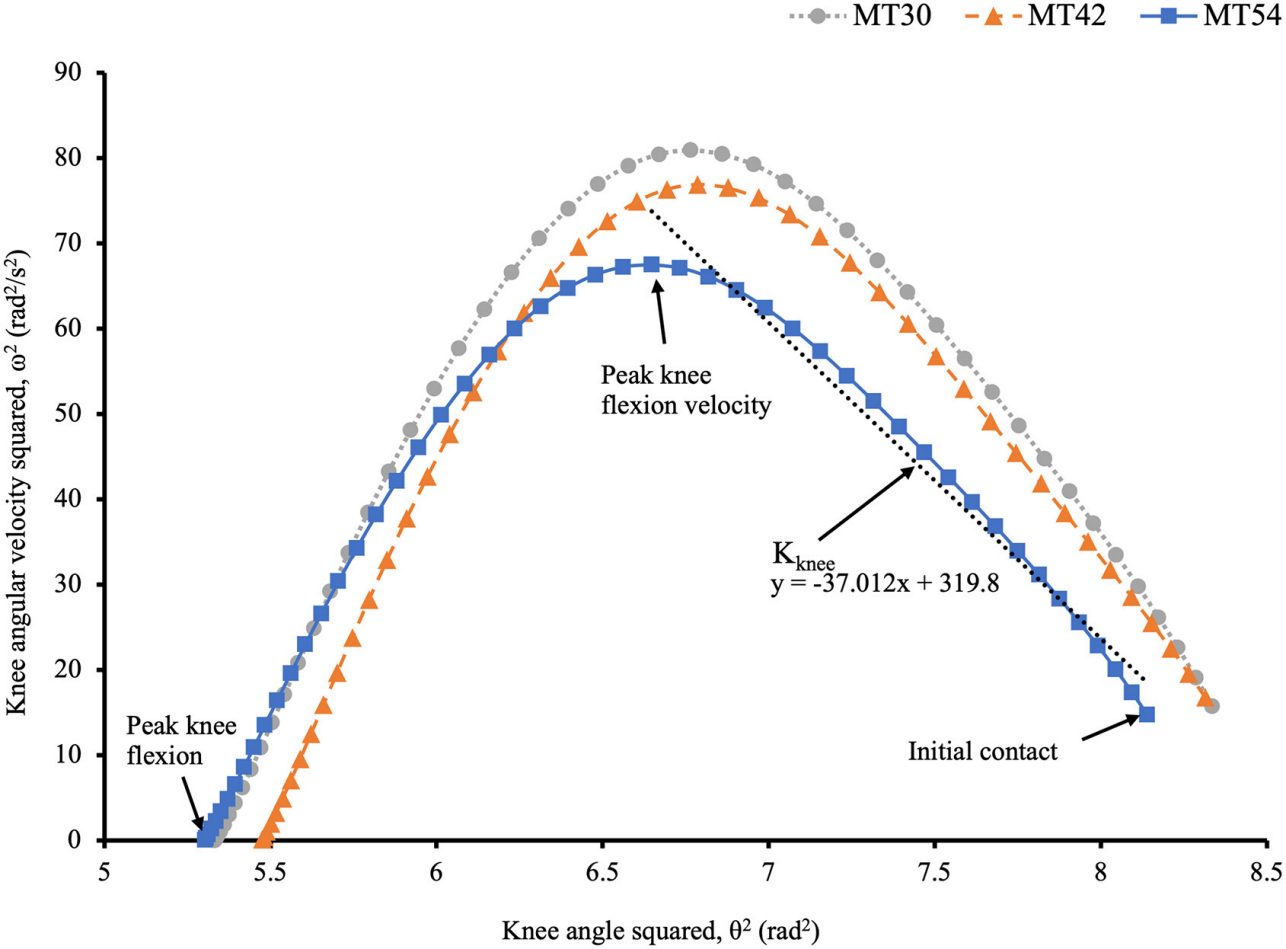
The relationship between ω^2^ and θ^2^ of knee joint on a typical subject trial between shoe conditions during the first half of stance. Notice the two distinct sub-phases with the upward part of the curve from IC representing the impact phase and the downward part represents a weight acceptance phase (not covered in this paper). Δ(ω^2^)/Δ(θ^2^) for knee joint stiffness calculation was determined as the slope of the regression line shown above (i.e., the coefficient of the regression equation) throughout 20–80% of the data points (the most linear portion) of the impact phase.

For variables following IC (i.e., during the impact phase), vertical loading rates in each shoe condition were calculated using a previously described method (Crowell and Davis, 2011). All successful trials showed a clear impact force peak in the vertical GRF during running. This allowed the vertical average loading rate (VALR) to be represented by the slope of the vertical GRF curve in a region between 20 and 80% from IC to the first vertical force impact peak, where vertical instantaneous loading rate (VILR) was the maximal slope of the vertical GRF curve between consecutive data points in that region. The impact phase for knee stiffness was defined from IC to peak knee flexion velocity and was determined using the method specified in Verheul et al. (2017). Knee joint stiffness (K_knee_) was calculated by the following equation:


(1) $$\begin{array}{l} \begin{array}{c} K_{knee}=\frac{I\cdot \frac{\Delta \left(\omega^{2}\right)}{\Delta \left(\theta^{2}\right)}}{ROM}\; \end{array} \end{array}$$

where *I* is the bodyweight of the participant multiplied by the squared thigh length (m·${\text{l}}_{\text{thigh}}^{2}$), ω is the knee angular velocity in rad/s, θ is the knee angle in radians, and ROM is the range of motion of knee joint in degrees during the impact phase. The thigh length was determined as the distance from the right greater trochanter to right lateral femoral epicondyle and the knee angle was defined as the angle between right thigh and shank segments. The relationship between knee angle squared (θ^2^) and knee angular velocity squared (ω^2^) was determined by fitting regression lines from 20 to 80% data points in the impact phase. Figure 2 illustrates an example calculation of (ω^2^)/(θ^2^) for the knee joint.

**Figure 3:**
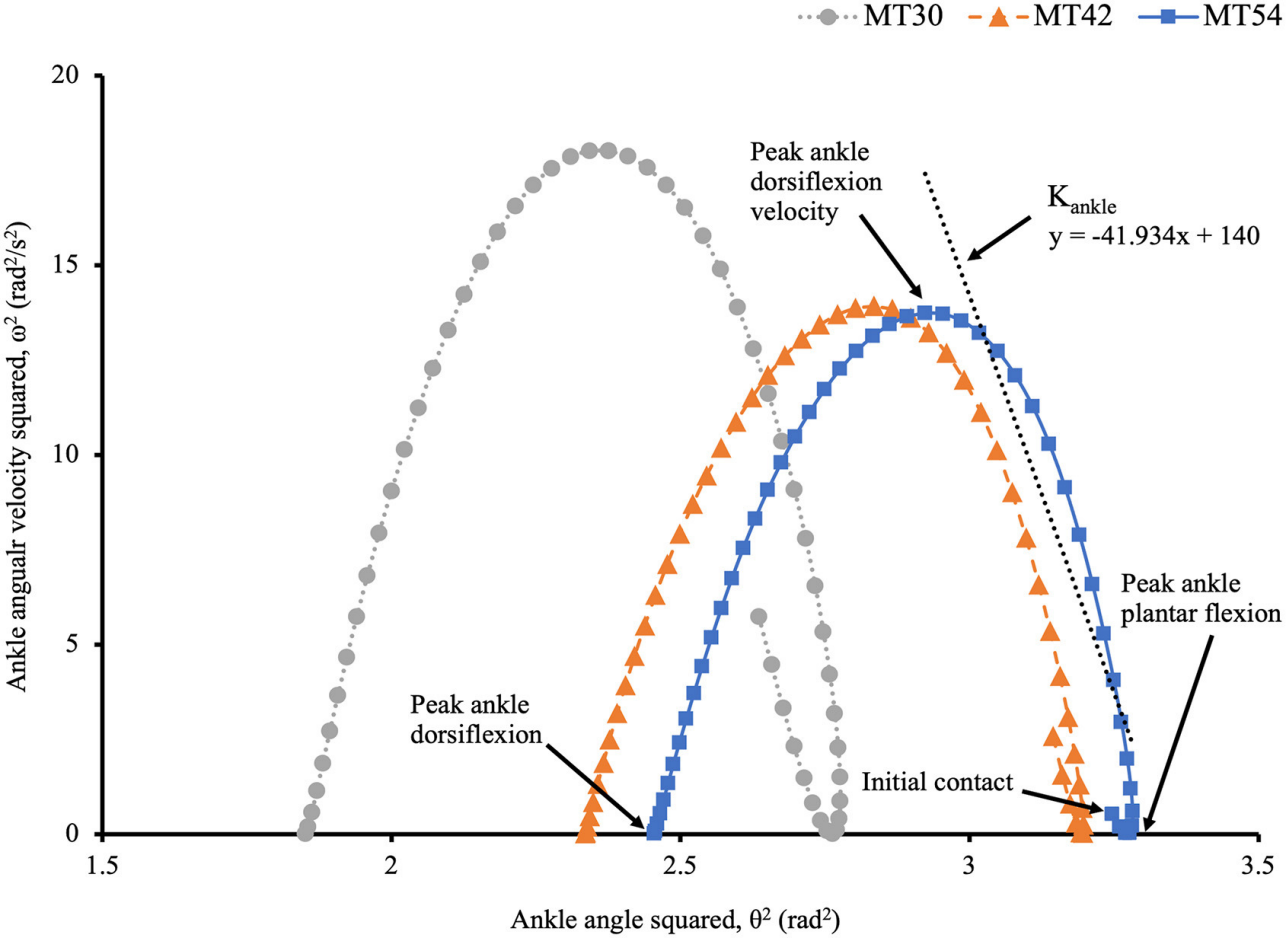
The relationship between ω^2^ and θ^2^ of ankle joint on a typical subject trial between shoe conditions during the first half of stance. Δ(ω^2^)/Δ(θ^2^) for ankle joint stiffness calculation was determined as the slope of the regression line shown above (i.e., the coefficient of the regression equation) throughout 20–80% of the data points (the most linear portion) of the impact phase.

Ankle joint stiffness (K_ankle_) was calculated using the same equation (1) but applied to the ankle joint, where *I* in that case is the bodyweight of the participant multiplied by the squared foot length (m·${\text{l}}_{\text{foot}}^{2}$) (Dutto and Braun, 2004). The foot length was determined as the distance from the right distal end of calcaneus to the right fifth metatarsal head, and the ankle angle was defined as the angle between right shank and foot segments. Ankle stiffness was not determined for the very early ground contact phase from IC to peak ankle plantar flexion, due to the significant variability in the relationship of (ω^2^)/(θ^2^) during this period across participants and shoe conditions. Hence, ROM of ankle joint in degrees was calculated from peak ankle plantar flexion to peak ankle dorsiflexion velocity for further stiffness calculation in this phase. The relationship between ankle angle squared (θ^2^) and ankle angular velocity squared (ω^2^) was determined by fitting regression lines from 20 to 80% data points in the impact phase. Figure 3 illustrates an example calculation of (ω^2^)/(θ^2^) for the ankle joint.

### Statistical Analysis

Each dependent variable in successful trials was averaged for each subject-shoe condition. A one-way repeated measures analysis of variance (ANOVA) was used to statistically examine the main effect of MT on the dependent variables of interest (following satisfactory tests for normal distribution of the data). An alpha level of *p* = 0.05 was set to detect statistical significance. *Post-hoc* pairwise comparisons with Bonferroni correction were conducted at the event of significant main effects. Partial eta-squared ($\eta_{\text{p}}^{2}$) was also calculated to further assess the effect size, where values of 0.01, 0.06, and 0.14 represent small, medium, and large effects, respectively (Cohen, 2013). All statistical analyses were performed using GraphPad Prism (GraphPad Software, CA, USA).

## Results

### Prior to IC

**Figure 4:**
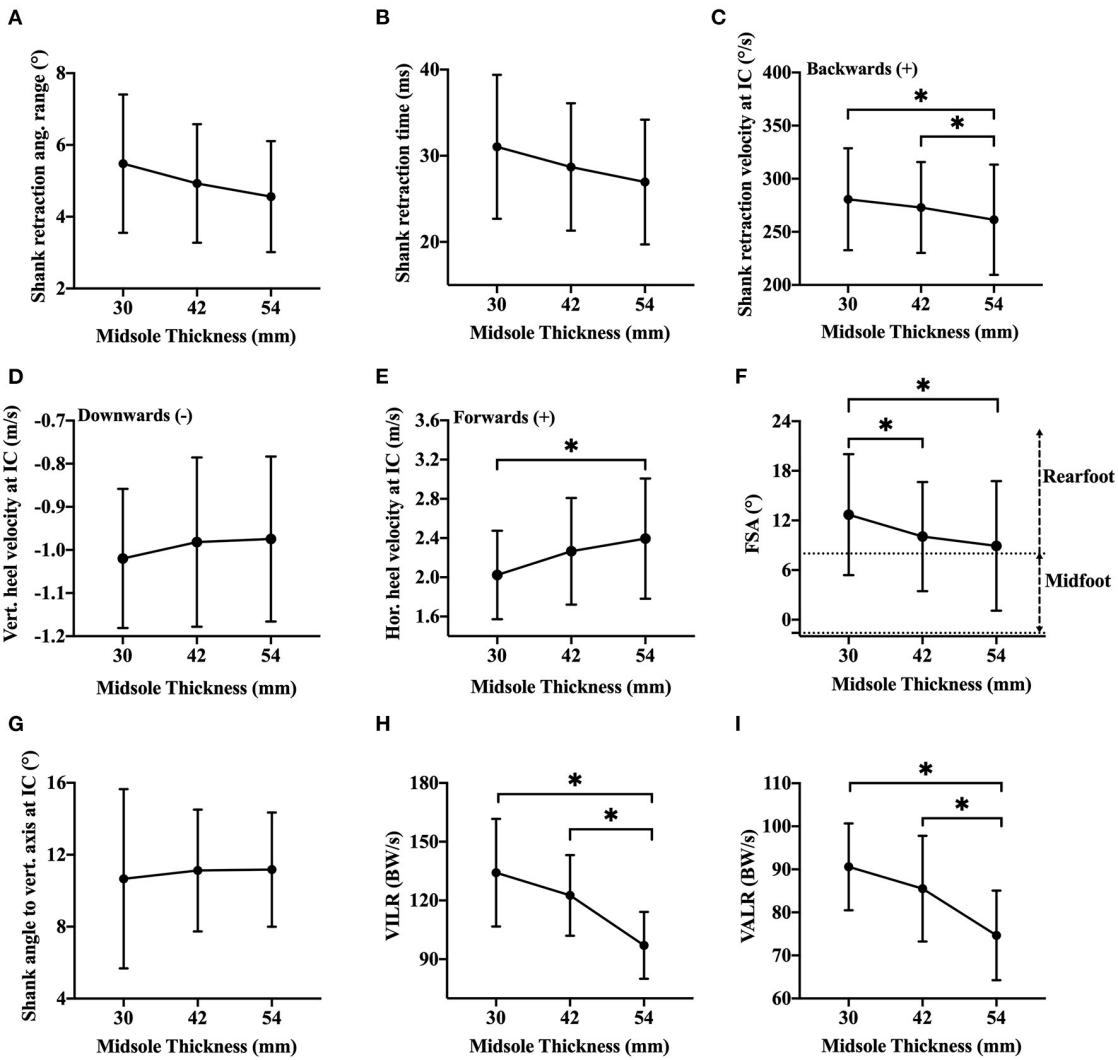
Mean ± SD of shank retraction angular range prior to IC **(A)**; shank retraction time prior to IC **(B)**; shank retraction angular velocity at IC **(C)**; vertical **(D)** and horizontal **(E)** heel velocity at IC; FSA **(F)**; shank angle to vertical axis at IC **(G)**; VILR **(H)** and VALR **(I)** in three shoe conditions, while *p < 0.05.

No significant effects of MT on both shank retraction angular range [*F*_(1,15)_ = 2.86, *p* = 0.10; Figure 4A] and retraction time [*F*_(1,14)_ = 2.93, *p* = 0.10; Figure 4B] were found.

### Instant of IC

For the dynamics of foot strike at IC, there was a significant effect of MT on both shank retraction angular velocity [*F*_(2,17)_ = 6.57, *p* < 0.05; Figure 4C] with a small effect size ($\eta_{\text{p}}^{2}$ = 0.029) and horizontal component of heel velocity [*F*_(2,18)_ = 8.63, *p* < 0.05; Figure 4E] with a medium effect size ($\eta_{\text{p}}^{2}$ = 0.081). *Post-hoc* comparisons indicated that IC shank retraction angular velocity in MT54 was significantly smaller than those using MT30 (mean difference = 19.3°/s) and MT42 (mean difference = 11.5°/s). Furthermore, MT54 caused significantly greater IC horizontal heel velocity (mean difference = 0.4 m/s) compared to MT30. No significant main effects of MT on vertical heel velocity were found [*F*_(1,14)_ = 0.85, *p* = 0.40; Figure 4D]. For lower leg posture at IC, MT showed a significant effect on FSA [*F*_(2,17)_ = 12.72, *p* < 0.05; Figure 4F], with a small effect size ($\eta_{\text{p}}^{2}$ = 0.049). *Post-hoc* comparisons revealed that using MT30 (12.7 ± 7.3°) resulted in a significantly higher FSA than MT42 (10.1 ± 6.6°) and MT54 (8.9 ± 7.8°), whereas no significant difference in FSA was found between MT42 and MT54 (*p* = 0.14). MT had no significant influence on shank angle to the vertical axis [*F*_(1,16)_ = 0.25, *p* = 0.71; Figure 4G].

### After IC

For the vertical loading rates, MT exhibited a significant effect on VILR [*F*_(2,17)_ = 39.30, *p* < 0.05; Figure 4H] with a large effect size ($\eta_{\text{p}}^{2}$ = 0.348). Lower VILR was found in MT54 (97.1 ± 17.1 BW/s), compared to MT30 (134.2 ± 27.5 BW/s) and MT42 (122.6 ± 20.6 BW/s), whereas no significant difference in VILR was found between MT30 and MT42 (*p* = 0.06). Similarly, MT showed a significant effect on VALR [*F*_(1,14)_ = 30.48, *p* < 0.05; Figure 4I] with a large effect size ($\eta_{\text{p}}^{2}$ = 0.286). *Post-hoc* comparisons indicated that MT54 (74.7 ± 10.4 BW/s) resulted in significantly lower VALR than MT30 (90.6 ± 10.1 BW/s) and MT42 (85.5 ± 12.3 BW/s). There was no significant difference in VALR between MT30 and MT42 (*p* = 0.27). Regarding joint stiffness of ankle and knee (Table 1) during the impact phase, MT showed a significant main effect on K_knee_ ($\eta_{\text{p}}^{2}$ = 0.181). *Post-hoc* comparison revealed MT54 resulted in significantly higher K_knee_ than MT42 and MT30. No significant difference in K_knee_ was observed between MT42 and MT30. Both knee range of motion and peak knee flexion velocity were not affected by the changes in MT. There was a significant effect of MT on both K_ankle_ ($\eta_{\text{p}}^{2}$ =0.464) and peak ankle dorsiflexion velocity ($\eta_{\text{p}}^{2}$ = 0.274). Significant increases in K_ankle_ were found from MT30 to MT42 and MT54, also from MT42 to MT54. Contrarily, significant decreases in peak ankle dorsiflexion velocity were found from MT30 to MT42 and MT54, also from MT42 to MT54.

**Table 1:** Mean ± SD of the joint stiffness, range of motion, and peak angular velocity of ankle and knee joint in the impact phase between shoe conditions.

	**MT30**	**MT42**	**MT54**	***F*-value**	***P*-value**
K_knee_ (Nm/°)	49.5, 18.0	53.5, 16.3	66.5, 13.7^†^^‡^	39.80	<0.001
ROM_knee_ (°)	14.0, 3.2	14.6, 2.5	13.8, 3.0	1.11	0.34
PKFV (°/s)	454.2, 83.0	453.1, 58.4	431.8, 69.3	1.70	0.21
K_ankle_ (Nm/°)	25.9, 6.0	30.8, 6.7^§^	40.2, 7.2^†^^‡^	93.08	<0.001
ROM_ankle_ (°)	9.5, 1.9	9.7, 2.5	9.1, 2.7	0.92	0.39
PADV (°/s)	285.6, 47.8	252.6, 32.9^§^	225.5, 42.93^†^^‡^	31.20	<0.001

† *Significant difference between MT54 and MT30*.

‡ *Significant difference between MT54 and MT42*.

§ *Significant difference between MT42 and MT30*.

*MT30, midsole thickness at 30 mm; MT42, midsole thickness at 42 mm; MT54, midsole thickness at 54 mm; K_knee_, knee joint stiffness; K_ankle_, ankle joint stiffness; PKFV, peak knee flexion velocity; PADV, peak ankle dorsiflexion velocity*.

## Discussion

The aim of this study was to re-examine biomechanical adaptations related to foot strike when running in footwear with different midsole cushioning thickness. Generally, across footwear conditions, the posture of the shank at initial ground contact was not significantly different, and there was a small but significant reduction (3–4 degrees) in the heel strike angle with increasing MT. There was a significant decrease in shank retraction velocity and increase in the horizontal velocity of the heel when MT was increased. Therefore, hypothesis one is accepted with increases in shoe MT associated with some adjustments in both lower limb posture (FSA) and dynamics. Hypothesis two was accepted as increases in midsole cushioning thickness significantly reduced vertical loading rates (impact severity) during running using two different calculation methods. Both impact phase stiffness setting of the ankle and knee joints were significantly increased with the ultra-cushioned midsole which partially supported the third hypothesis. Collectively, the findings point toward the benefit of monitoring adjustments in the initial conditions of ground contact, in terms of both lower limb posture and dynamics, to understand foot strike mechanics, and the initial stiffness setting of the joints when running in different footwear conditions. The presence or lack of active impact-moderating behavior is likely to be informative from an injury prevention perspective.

**Figure 5:**
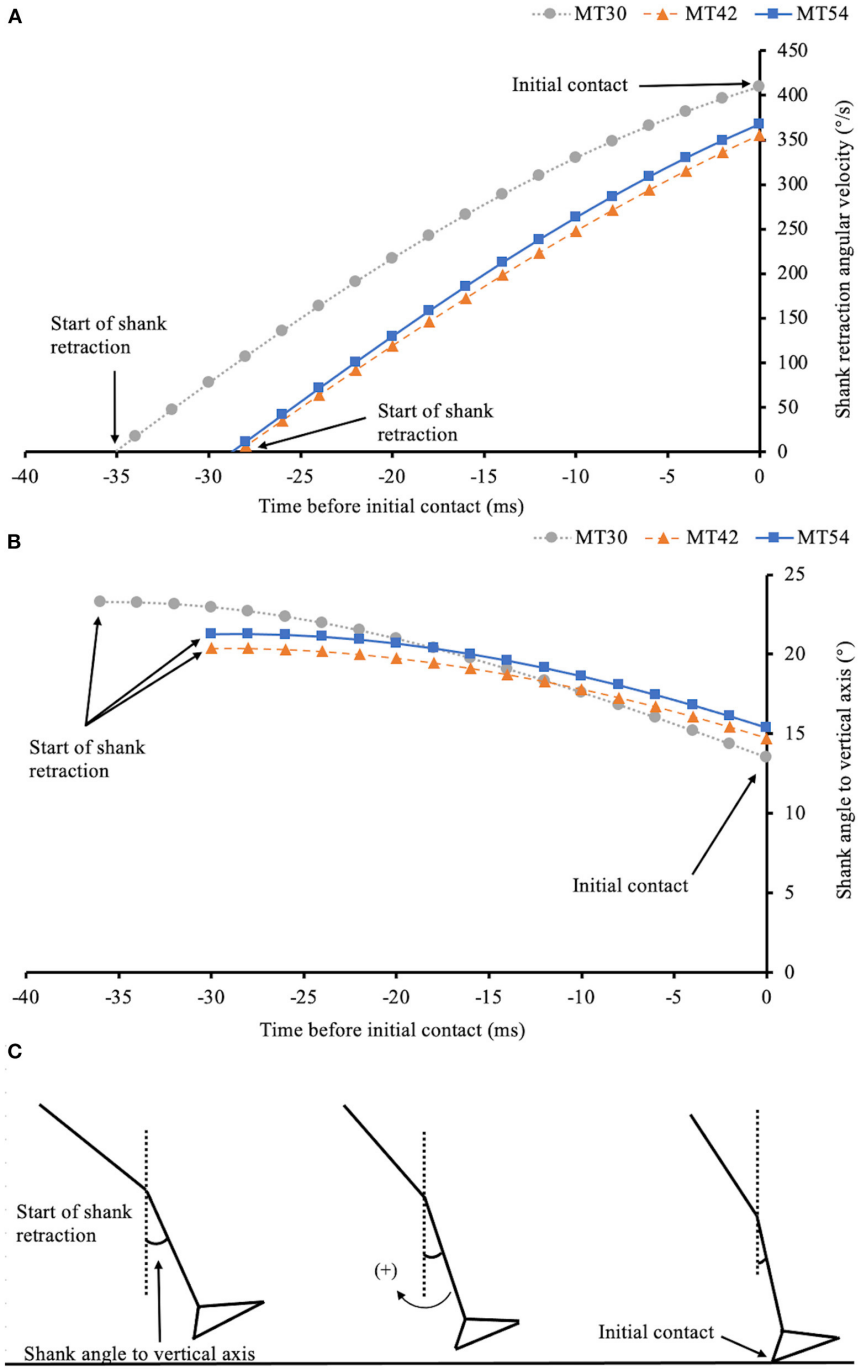
Retraction angular velocity **(A)** and angular displacement **(B)** of the shank prior to IC for a typical trial in each shoe condition for one of the subjects. The schematic stick figure **(C)** illustrates the evolution of shank retraction. Notice that an earlier initiation of retraction motion in the thinner shoe midsole condition (i.e., MT30) does not influence much the posture of the shank at initial ground contact but leads to an increased retraction angular velocity of the shank at IC. The same convention of shank retraction angular velocity applies to Figure 4C.

Regarding the retraction of the shank prior to IC, all the participants started rotating the shank backward (i.e., flexing their knee) before landing which accords with the notion of swing-leg retraction (Seyfarth et al., 2003). The amount of shank retraction was typically around 5–6 degrees during the last 30 ms before contact which closely agrees with previous work (Seyfarth et al., 2003). This late swing phase movement strategy employed by the subjects in this study has been deemed to reduce impact severity of the foot-ground collision and enhance the stability of running (De Wit et al., 2000; Herr et al., 2002). A decreasing trend of both shank retraction angular range and retraction time can be observed with increased MT, with the shank tending to begin rotating backward earlier in the thinner shoe condition (refer to Figure 5 for a representative subject) but statistical significance did not met (refer to Figures 4A,B). Figure 5 illustrates that, despite slightly different movement trajectories of the shank prior to landing, the angle or posture of the shank at the instant of contact was maintained. For the group of subjects, we found that shank angle to the vertical at IC was not influenced by MT differences. This agrees with previous research (Seyfarth et al., 2003) which stated that the swing-leg retraction was a strategy used to select an “angle of attack” that sustained a desired movement pattern. Although the IC shank posture was unchanged, IC shank retraction angular velocity was found to be higher in the shoes with a thinner midsole (Figures 4C, 5). This higher shank retraction angular velocity was associated with a reduced horizontal component of IC heel velocity (Figure 4E). This finding is in accordance with a previous study comparing the heel velocity between barefoot and shod running conditions (De Wit et al., 2000). Similar to the findings of this current study, those authors (De Wit et al., 2000) found that the vertical component of heel velocity at landing was unchanged between shod and unshod conditions, and adjustments in the dynamics of landing were restricted to the horizontal component. Previous studies indicated that runners might adopt a decelerated vertical heel velocity at landing to reduce the vertical momentum of the heel in less cushioned shoes (Gerritsen et al., 1995), thereby reducing the severity of impact. But interestingly, the active adjustments in landing foot velocity appear mostly in the horizontal direction. Further work is needed to determine the direct association between adjustments in shank retraction velocity and the resulting foot velocity at the instant of landing to moderate the severity of impact during running.

Our findings on FSA were inconsistent with most of the previous studies which indicated that footwear with increased MT would promote a more rearfoot strike (Squadrone and Gallozzi, 2009; Horvais and Samozino, 2013; Squadrone et al., 2015; Yang et al., 2019), as this alteration would allow more deformation of cushioning materials and consequently reduce the discomfort caused by the impact from foot-ground collision (Lieberman et al., 2010). This inconsistency could be explained by two facts: (1) the increased MT in this study inevitably reduced the heel to toe drop of shoes (i.e., 7 mm for MT30, 5 mm for MT42, and 3 mm for MT54). Heel to toe drop is the difference between heel stack height and toe stack height. Previous literature has demonstrated that runners would progress toward a decreased FSA when running in the shoes with heel to toe drop close to zero (Chambon et al., 2015). (2) The footwear MT tested in this study, even for the thinnest one (i.e., MT30), was exaggeratedly thicker than normal running shoes. The increased MT was reported to significantly increase the peak GRF in both medial–lateral and anterior–posterior directions, which would be attributable to the landing instability (Robbins et al., 1994; Zhang and Li, 2016). Therefore, in MT42 and MT54, runners may exhibit a lower FSA (i.e., less ankle dorsiflexion) to lower the center of mass of the foot segment and meanwhile to gain more contact area to the ground for foot stabilization during landing. However, although the results of FSA showed a trend of decreasing in MT42 and MT54 compared to MT30, participants did not transit to a non-rearfoot strike pattern in thicker shoe conditions, and there was only a small effect size ($\eta_{\text{p}}^{2}$ = 0.049) of MT on FSA. Such results were similar to the findings reported by Law et al. (2019), which showed the running shoes with thick midsole (29 mm in that study) no longer led to an increased FSA, compared to the relatively thinner one (25 mm in that study). In this study, rather than switching to a non-rearfoot strike pattern, participants appeared to adjust the shank retraction angular velocity and horizontal component of heel velocity at IC. However, although the categorical heel strike pattern remained the same, there was a continuous shift in heel strike angle with changing MT (refer to Figure 4F) which is in agreement with the work of Gruber et al. (2021). In terms of the results after IC, our findings on vertical loading rates matched with the second hypothesis. Contrary to studies that reported thinner midsole would cause lower vertical loading rates (Pollard et al., 2018), a clear trend of decreased VALR and VILR was found in MT54, compared to MT30 and MT42. It is suspected that this conflicting result could be attributed to the runners in previous studies adopted a non-rearfoot strike pattern with thinner midsole and consequently caused lower vertical loading rates (Lieberman et al., 2010), whereas participants in this study maintained heel-first strike patterns across all shoe conditions. Lower vertical loading rates could be expected in the thicker midsole condition (i.e., MT54) since the extra-cushioned heel allows more vertical deformation of the midsole materials and hence dampens impact loading from the foot-ground collision (Lieberman, 2012; Gruber et al., 2021), although a previous study indicated that the MT beyond 25 mm might not further reduce the vertical loading rates (Law et al., 2019). This could be explained by the spectrum of MT tested, as the maximum MT tested in that previous study only reached 29 mm, which is similar to the thinnest midsole tested in this study (i.e., 30 mm). The exceptionally increased MT in this study (i.e., MT54) might be beneficial for runners in reducing the development of specific running injuries caused by the impact from foot-ground collision. Many studies have suggested that increased VALR and VILR may be linked with higher risks of running-related injuries (Milner et al., 2006; Pohl et al., 2009; Cheung and Davis, 2011), but meta-analyses indicate that although some injury types such as stress fractures may have some association to loading rates, more prospective studies are needed to establish the relationship between impact force loading rate and lower limb injury (van der Worp et al., 2016).

For the impact phase lower limb joint stiffness, the large increase in K_knee_ in MT54 may be partly attributed to knee joint stabilization, as we suspected that the extremely cushioned midsole could cause the instability of lower limbs. Participants would have been exposed to running in such a thick-soled shoe for the first time, and previous research suggested that the neuromuscular system would increase activation levels of muscles around the knee and stiffen the joint as the part of the impedance control process (Burdet et al., 2001; Franklin et al., 2004). Although joint stability parameters were not directly measured in this study, the participants likely adapted to the exaggerated MT by controlling the movement of knee joint to maintain the knee joint stability (Baltich et al., 2015). *Post-hoc* inspection of the force and motion data revealed that in some subjects, there were indications of increased lower limb movements and oscillations in the frontal plane during early stance while wearing the thickest midsole shoes. Larger and more rapid deviations in the center of pressure and larger peaks in the medial–lateral GRF were evident. There was a tendency for the MT54 shoe to be associated with lower peak knee flexion velocities in this study but knee ROM during the impact phase remained the same as the other shoes. The stiffer knee joint in MT54 means that the knee was attenuating less load, which may result in the development of lower back pain for runners (Hamill et al., 2009). Our findings also indicated that the increased MT was associated with greater K_ankle_, which suggests the ankle would transmit more load as well. For the ankle joint, the increased stiffness with MT was linked to a significantly reduced peak ankle dorsiflexion velocity (refer to Table 1). Again, this could possibly be partly due to elevated muscle activation levels around the ankle, to stiffen the joint at landing and during the impact phase alongside changing dynamic conditions (rate of joint flexion) at that time.

There are several limitations to be acknowledged in this study. First, the mass across the three shoe conditions was not identical and that may partly influence the changing active kinematic adaptations at ground contact seen in this study. Second, 5 min of familiarization time might be inadequate for participants to exhibit a distinct alteration in foot landing strategies in the different footwear conditions. Further studies should allow longer familiarization time or examine the long-term effect of running shoes with different MT. It remains to be seen what adjustments in landing kinematics (dynamics and posture at IC) may develop with more prolonged usage of the different shoe conditions and in runners with habitual non-rearfoot strike patterns. It is necessary to replicate these results in larger longitudinal studies to determine whether the apparent impact moderating strategies are maintained. Third, it is possible that some of active pre-landing and landing adaptations to running in more cushioned shoes might be masked by subtle targeting effects during the current protocol that involved contacting a force platform in the middle of a runway. Improved consistency in landing strategies in a specific footwear condition might be revealed during treadmill running at a controlled running speed (Seyfarth et al., 2003). Finally, the kinematic approach used to determine joint stiffness in a distinct, early impact phase of running deviates from most investigations that use the relationship between joint moment and joint angle during the entire first half of stance to estimate adjustments in joint stiffness. Verheul et al. (2017) employed the same calculation method for joint stiffness and reported that high-mileage runners exhibited higher knee joint stiffness during the impact phase compared with low-mileage runners, and they also reported an increased knee joint stiffness when running with higher speed. The findings of this study are applicable to the tested running speed only. Hence, it is worthwhile to investigate whether the ultra-cushioned footwear would be a passive strategy to modify the lower limb joint stiffness for runners with a different running mileage and speed. The different computational methods will influence estimates of joint stiffness (acknowledged by Gruber et al., 2021) and comparisons between studies are then difficult. However, it becomes more accepted that the joint stiffness during the first half of running stance is not adequately modeled as a linear spring with constant stiffness (e.g., Nigro et al., 2021), but splitting that period into at least two phases (an impact phase and a weight acceptance phase) can perhaps allow linear assumptions to be reasonable (Verheul et al., 2017).

## Conclusion

This study examined adjustments in biomechanical parameters associated with foot strike when running in the shoes with different midsole cushioning thickness characteristics. Dynamic aspects of the initial conditions of landing and an impact phase stiffness measurement were added to typical measurements of foot strike mechanics that might be related to the risk of injury. Vertical force loading rates were higher as MT decreased in rearfoot strikers, whereas the dynamics of landing were modified to increase retraction velocity of the shank just prior to landing and decrease horizontal heel velocity. In contrast, the posture of the foot and lower leg at IC were not influenced much by MT changes. Both knee and ankle impact phase stiffness decreased with the thinner less cushioned shoe conditions, and this reveals the need to investigate further the interaction between shoe cushioning characteristics and active adjustments in foot strike mechanics during running in terms of how they collectively influence joint stiffness regulation. The dynamic measurements presented in this paper allow researchers to monitor impact moderating behavior more comprehensively during running and the possible associated changes in running-related injury risk.

## Data Availability Statement

The raw data supporting the conclusions of this article will be made available by the authors, without undue reservation.

## Ethics Statement

The studies involving human participants were reviewed and approved by Liverpool John Moores University Ethics Committee. The patients/participants provided their written informed consent to participate in this study.

## Author Contributions

ZZ contributed to methodology, data collection, data analysis, investigation, and writing original draft and editing. ML contributed to conceptualization, methodology, supervision, reviewing, and editing. All authors contributed to the article and approved the submitted version.

## Funding

Footwear for this study was provided by New Balance Inc.

## Author Disclaimer

Any opinions, findings, conclusions, and recommendations included in this study do not represent the views of New Balance Inc., U.S.A.

## Conflict of Interest

This study received funding from New Balance Inc., USA. The funder had the following involvement with the study: they provided the systematically modified footwear but had no involvement in the study design, collection, analysis, interpretation of data, the writing of this article, or the decision to submit it for publication. The authors declare that the research was conducted in the absence of any commercial or financial relationships that could be construed as a potential conflict of interest.

## Publisher's Note

All claims expressed in this article are solely those of the authors and do not necessarily represent those of their affiliated organizations, or those of the publisher, the editors and the reviewers. Any product that may be evaluated in this article, or claim that may be made by its manufacturer, is not guaranteed or endorsed by the publisher.
